# The Promiscuity of Squalene Synthase-Like Enzyme: Dehydrosqualene Synthase, a Natural Squalene Hyperproducer?

**DOI:** 10.1021/acs.jafc.3c05770

**Published:** 2024-02-05

**Authors:** Zheng Guan, Yafeng Song, Marcel de Vries, Hjalmar Permentier, Pieter Tepper, Ronald van Merkerk, Rita Setroikromo, Wim J. Quax

**Affiliations:** † Department of Chemical and Pharmaceutical Biology, Groningen Research Institute of Pharmacy, University of Groningen, Groningen9713 AV, The Netherlands; ‡ Interfaculty Mass Spectrometry Center, Groningen Research Institute of Pharmacy, University of Groningen, Groningen9713 AV, The Netherlands; § Guangdong Provincial Key Laboratory of Microbial Culture Collection and Application, State Key Laboratory of Applied Microbiology Southern China, Institute of Microbiology, Guangdong Academy of Sciences, Guangzhou510070, China

**Keywords:** CrtM, SQS, squalene, enzyme promiscuity, terpenoid

## Abstract

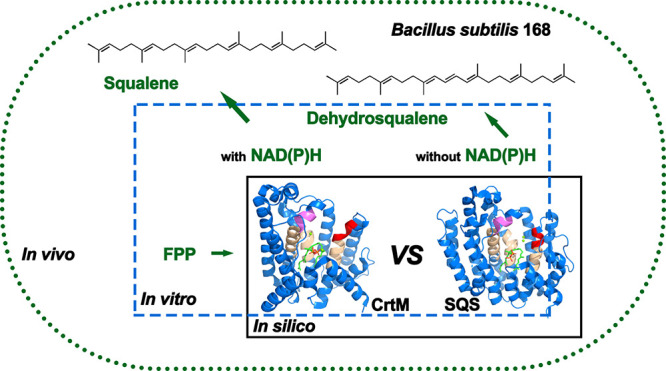

Dehydrosqualene synthase (CrtM), as a squalene synthase-like enzyme from *Staphylococcus aureus*, can naturally utilize farnesyl diphosphate to produce dehydrosqualene (C_30_H_48_). However, no study has documented the natural production of squalene (C_30_H_50_) by CrtM. Here, based on an HPLC-Q-Orbitrap-MS/MS study, we report that the expression of *crtM in vitro* or in *Bacillus subtilis* 168 both results in the output of squalene, dehydrosqualene, and phytoene (C_40_H_64_). Notably, wild-type CrtM exhibits a significantly higher squalene yield compared to squalene synthase (SQS) from *Bacillus megaterium* with an approximately 2.4-fold increase. Moreover, the examination of presqualene diphosphate’s stereostructures in both CrtM and SQS enzymes provides further understanding into the presence of multiple identified terpenoids. In summary, this study not only provides insights into the promiscuity demonstrated by squalene synthase-like enzymes but also highlights a new strategy of utilizing CrtM as a potential replacement for SQS in cell factories, thereby enhancing squalene production.

## 1 Introduction

Squalene, a well-known triterpene with diverse bioactivities, plays a pivotal role in the fields of food, medicine, and cosmetics.^1−3^ Historically, squalene has mainly been produced from agriculture, specifically sharks in the fishing industry and olives in the cultivation sector.^4^ However, in recent years, there has been a significant shift toward biosynthetic alternatives, gradually replacing traditional sources and becoming key contributors to large-scale squalene production. Notably, yeast-based biosynthesis has made impressive progress, exemplified by noteworthy investigations led by Cui et al.^5^ and Hong et al.,^6^ wherein strains like *Pseudozyma* sp. SD301 and *Pseudozyma* sp. JCC207 have achieved astonishing squalene yields of up to 2.445 g/L and 340.52 mg/L, respectively, without any genetic modifications. Nevertheless, despite these remarkable advancements, the biosynthesis of squalene still mainly relies on the head-to-head condensation of two molecules of farnesyl diphosphate (FPP) catalyzed by squalene synthase (SQS).

Unexpectedly, in our pilot experiment, we made an interesting discovery that dehydrosqualene synthase (CrtM) is also capable of producing squalene. This has sparked speculation about the potential of CrtM to serve as a replacement for SQS in cellular factories and opened up new strategies for squalene biosynthesis.

As is well known, CrtM is a prominent representative of prenyltransferases involved in carotenoid biosynthesis. Operating as a multifunctional enzyme, CrtM facilitates a two-step reaction encompassing the conversion of FPP to presqualene diphosphate (PSPP), followed by dehydrosqualene synthesis.^7−10^ Over the past two decades, researchers have noted the striking functional, evolutionary, and structural resemblance between CrtM and SQS,^7,11^ classifying it as a squalene synthase-like (SSL) enzyme. Consequently, CrtM has attracted attention in screenings for SQS inhibitors targeting the virulence of *Staphylococcus aureus*,^7,12−14^ as well as stimulating interest in synthetic biology, food science, and medicine. Umeno et al.^8^ reported in 2002 that natural CrtM exhibited limited capacity to utilize geranylgeranyl diphosphate (GGPP) for synthesizing the C40 carotenoid backbone, suggesting flexibility within its active site. Furthermore, it was discovered that CrtM could employ GGPP to produce C40 carotenoids like phytoene. Subsequent investigations have leveraged the flexibility of CrtM, directly mutating its active site, thereby enabling the production of diverse long-chain terpenoids alongside dehydrosqualene.^15−18^ However, the synthesis of squalene by CrtM has not been reported to date.

Here, we report the serendipity when doing the follow-up study of our carotenoid production research.^19^ It was found that CrtM, while studying its expression in *Bacillus subtilis*, has the ability to produce squalene, as well as dehydrosqualene.

In this regard, we have performed the following research *in vivo*, *in vitro*, and *in silico* to verify that the SSL enzymes have catalytic promiscuity and can synthesize a series of terpenoids. We show that CrtM derived from *S. aureus* is a natural squalene producer and potentially can be created as a squalene hyperproducer.

## 2 Materials and Methods

### 2.1 Materials and Reagents

Squalene standard (≥98%), FPP (≥95%), and GGPP (≥95%) were purchased from Sigma (Sigma-Aldrich, USA). Methanol, acetone, ethyl acetate, acetonitrile, d-xylose, and 2-propanol were purchased from Merck (Merck, Germany). Enzymes were obtained from Thermo (Thermo Fisher Scientific, USA). Other culture media components were purchased from Duchefa (Duchefa Biochemie, The Netherlands).

*Escherichia coli* DH5α and *B. subtilis* 168 (Novagen, The Netherlands) were used for gene cloning and protein expression, respectively. *CrtM* and *crtN* genes were obtained from *S. aureus*. The *SQS* gene was obtained from *Bacillus megaterium* (GenBank: ADF40697.1). All the plasmids and strains constructed in this study are listed in Table S1. The primers (Table S2) used in this study were synthesized by Eurofins (Eurofins Scientific, The Netherlands).

### 2.2 *In Vivo* Study

As a coincidental finding, during the initial phase of our investigation,^19^*B. subtilis* 168 strains containing *crtM* and *crtN* genes sourced from *S. aureus* were employed to examine carotenoid production. Subsequent detection of unforeseen signals prompted an investigation into quantifying squalene production mediated by CrtM in *B. subtilis* 168. To this end, *crtN* was knocked out, resulting in a *B. subtilis* strain exclusively expressing *crtM* (SCrtM). Thereafter, *B. subtilis* harboring the *SQS* gene from *Bacillus megaterium* (BSQS) was employed as a comparative reference (Table S1). Meanwhile, the study also investigated the impact of different culture media on the CrtM strain. Moreover, a comparison between the CrtM strain and an upstream-strengthened strain with both *crtM* and *dxs* (Dxs-SCrtM, Table S1) was made as a supplement. Each group had six independent inoculations for growing the culture.

The mentioned plasmids were constructed as follows. The pHY300PLK plasmid containing the *crtM* and *crtN* genes from *S. aureus* was constructed and transformed into *B. subtilis* 168 strains following previously reported methods.^19,20^ The *B. subtilis* strains with *crtM* or *SQS* in the comparison were both carried by plasmid pHY300PLK with the same constitutive promoter.^20^ The pHY300PLK plasmid carrying only the *crtM* gene was constructed by knockout of the *crtN* gene from the aforementioned plasmid. *CrtN* was cut out with restriction enzymes XbaI and NheI, and the plasmid was religated by T4 ligase. Additionally, the MEP pathway gene *dxs* was extracted from the previously constructed pHB201-SDFH plasmid, as reported by Xue et al.^19^ using XbaI and SpeI restriction enzymes, and then ligated with linearized pHCMC04G (also digested with XbaI and SpeI) via T4 ligase, resulting in the construction of pHCMC04G-S with a xylose-inducible promoter PxylA.^21^ The *SQS* gene was obtained from *B. megaterium* (GenBank: ADF40697.1), synthesized, and codon optimized to *B. subtilis* 168 (Eurofins, Netherlands). The prolonged overlap extension polymerase chain reaction (POE–PCR) method described by You et al.^22^ was used to construct the plasmid, where the ribosome binding site (RBS) and spacer (AAAGGGGG) were added at the N-terminus of the BSQS, and a 6 × His-tag (CATCATCATCATCAT-CAT) was placed upstream of the stop codon. The POE–PCR product was directly transformed into *E. coli*, and all strains were plated on LB agar plates containing the appropriate antibiotics. Positive colony-PCR results were subsequently confirmed by sequencing (Macrogen, Netherlands).

Strains were precultured at 37 °C overnight and inoculated at a ratio of 1:100 (v/v) into 25 mL of tryptic soy broth (TSB) (1.7% tryptone, 0.3% soytone, 0.25% dextrose, 0.5% NaCl, 0.25% K_2_HPO_4_) or 2SR media (5% yeast extract, 3% tryptone, and 0.3% K_2_HPO_4_) in a flask for fermentation, six repeats per strain. d-Xylose was added to a final concentration of 1% (m/v) when necessary to induce *dxs* expression once the bacteria’s OD_600_ reached around 0.5–0.6; the fermentation starting point of the other strains are also at the same OD_600_. Then, bacterial cells were harvested after 48 h of fermentation at 37 °C and 200 rpm. Antibiotics were added where appropriate (ampicillin at 100 μg/mL for *E. coli*, chloramphenicol at 5 μg/mL, and tetracycline at 20 μg/mL for *B. subtilis*).

Finally, bacterial cells were harvested and extracted as we described in a previous study.^23^ For samples that require determination of triterpenoid contents, the internal standard method was employed by supplementing squalene standard at a concentration equivalent to 40 μg/L of culture. The final extracts were dissolved in 250 μL of isopropanol–acetonitrile (7:3, v/v). Before injection into the HPLC-Q-Orbitrap-MS/MS system, all samples were filtered through a 0.22 μm membrane.

### 2.3 *In Vitro* Experiment

The expression and purification of the SCrtM protein were performed using established methodologies, as described in a previous literature.^7^ Subsequently, to assess its promiscuity in utilizing FPP and GGPP with NAD(P)H as cofactor for the biosynthesis of squalene and hydrogenated phytoene (C_40_H_66_), experimental protocols inspired by the *in vitro* test employed in SQS studies^24,25^ were implemented to obtain putative reaction products. The concentrations of the FPP, GGPP, and NAD(P)H added for the reaction are 20, 20, and 0.5 mM, respectively. After 15 min of reaction under 37 °C, we terminated the reaction.

The products were then extracted by 3 mL of petroleum and discarded in the frozen aqueous phase. After the organic layer was washed with 2 mL of Milli-Q water and the frozen aqueous phase was removed again, the samples were dried using nitrogen, dissolved in 250 μL of isopropanol–acetonitrile (7:3, v/v), and filtered through a 0.22 μm membrane before the HPLC-Q-Orbitrap-MS/MS detection.

### 2.4 HPLC-Q-Orbitrap-MS/MS Detection

The identification of the terpenes was performed by using an Agilent 1200 HPLC system equipped with a reversed-phase LichroCart C-8 guard column (5 μm, 4 × 4 mm, Merck, Darmstadt, Germany) or other C8 guard columns, and a Q Exactive Orbitrap mass spectrometer (Thermo Fisher Scientific, Bremen, Germany). The mobile phase was composed of phase A, isopropanol–acetonitrile (7:3, v/v), and phase B, water–acetonitrile (7:3, v/v). The constant flow rate was 0.6 mL/min, the injection volume was 25 μL, and gradient elution conditions were as follows: 0–3 min, 0–100% A; 3–4 min, 100% A; 4–6 min, 100–0% A. The mass spectrometer was adopted to acquire tandem mass spectrometry (MS/MS) on high resolution with a parallel reaction monitoring (PRM) mode in the control of the acquisition software (Xcalibur, Thermo Fisher Scientific, Bremen, Germany). The atmospheric pressure chemical ionization (APCI) source conditions included 40 Arb of sheath gas flow rate, 5 Arb of aux gas flow rate, 300 °C of capillary temperature, 140,000 of full MS resolution, collision energy as 30 or 35 in NCE mode, and 3.0 kV (positive) of spray voltage. The full scan was measured between *m*/*z* 150 and *m*/*z* 1500 in positive ion mode. A linear standard curve was plotted using a series dilutions of standards with known concentrations (Table S3). The contents of the analytes were calculated back to microgram per liter culture (μg/L).

### 2.5 *In Silico* Analysis

The protein sequence comparisons were performed through the EMBOSS Needle platform (https://www.ebi.ac.uk/Tools/psa/emboss_needle/). The crystal structures of enzymes were downloaded from the protein data bank (https://www.rcsb.org/: SCrtM, PDB ID 3NPR; human squalene synthase (HSQS), PDB ID 3WEH).^26,27^ The structures of ligands (https://pubchem.ncbi.nlm.nih.gov/) were retrieved from the PubChem database. The stereostructure of BSQS was modeled using HHpred, HHpred-TemplateSelection, and MODELER^28−30^ based on its amino acid sequences in FASTA format (https://www.ncbi.nlm.nih.gov/, GenBank: ADF40697.1). CB-Dock2,^31^ an online server for protein–ligand blind docking, and a visualization tool PyMOL (http://www.PyMOL.org/), was employed to prepare and visualize the results of molecular docking. The dockings were verified by AutoDock Vina,^32^ the docking engine of the CB-Dock2. The binding site for protein–ligand interaction and the related information such as the grid parameter *x*, *y*, and *z* coordinate values, grid values, affinities, and the contact residues are provided in Table S4.

## 3 Results and Discussion

### 3.1 Identify Squalene, Dehydrosqualene, and Phytoene Both *In Vitro* and *In Vivo*

In order to ascertain the promiscuity of CrtM in biosynthesis while excluding interference from other factors, investigations were conducted to evaluate the capability of the purified CrtM protein to utilize FPP, GGPP, and NAD(P)H for the production of specific products. HPLC-Q-Orbitrap-MS/MS detection has been employed to confirm these products both *in vitro* and *in vivo*.

**Figure 1:**
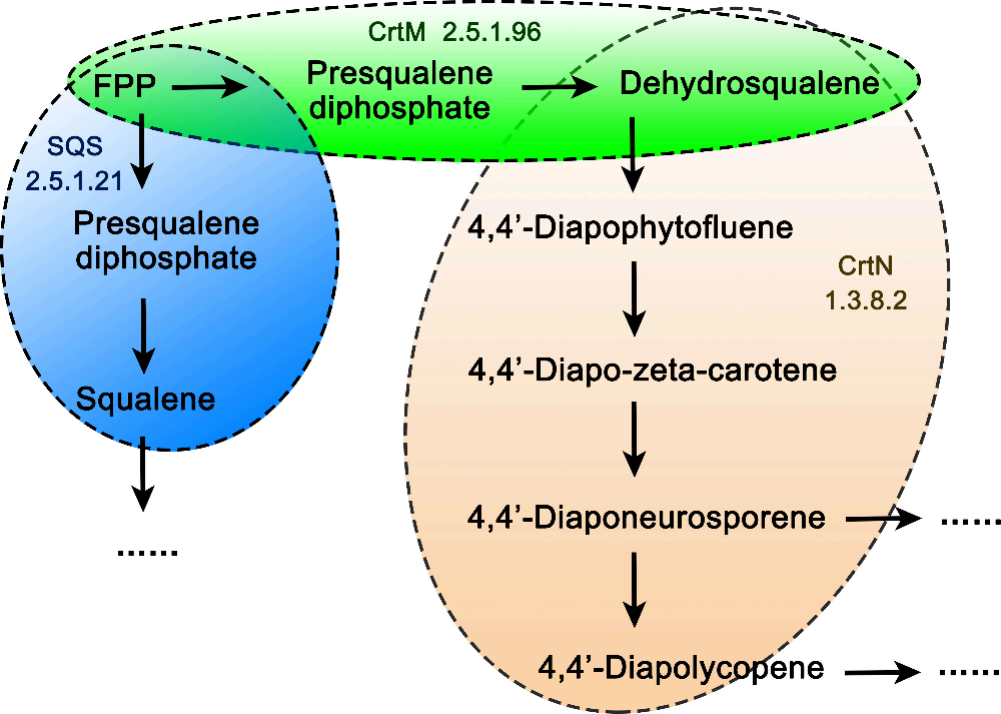
Related pathways of SQS, CrtM, and CrtN.

It is well known that enzymes are usually relatively plastic and can accept a variety of both natural and unnatural substrates.^8,33^ Given the dual substrate utilization capability of CrtM, encompassing both FPP and GGPP,^8^ along with its shared first-step reaction and intermediate product PSPP with SQS (as illustrated in Figure 1), the squalene, dehydrosqualene, phytoene, and hydrogenated phytoene signals were investigated in the *in vitro* study, as well as in the SCrtM and BSQS strains.

**Figure 2:**
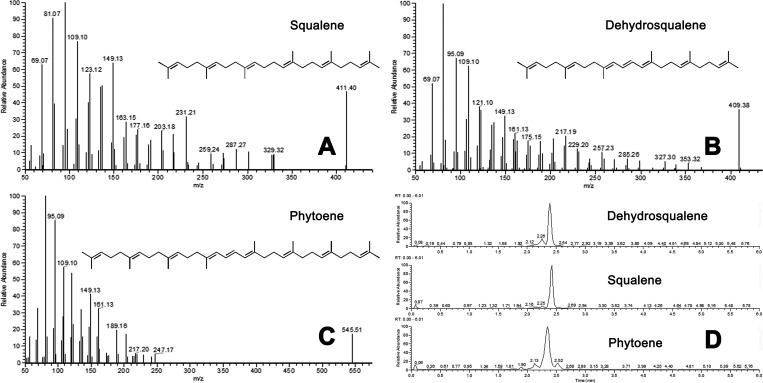
Identification of squalene, dehydrosqualene, and phytoene from *B. subtilis* SCrtM strain and BSQS strain by HPLC-Q-Orbitrap-MS/MS. (A) Example MS/MS spectra for squalene identification (extracted from SCrtM strain). (B) Example MS/MS spectra for dehydrosqualene identification (extracted from BSQS strain). (C) Example MS/MS spectra for phytoene identification (extracted from BSQS strain). (D) Example extracted ion chromatogram of dehydrosqualene, squalene, and phytoene (extracted from SCrtM strain).

Ultimately, squalene was identified by comparing with the squalene standard, and the other three compounds were tentatively identified (Figure 2, *m*/*z* error ≤2 ppm). The (+) APCI/Orbitrap mass spectra showed pseudomolecular ions ([M + H]^+^) at *m*/*z* 411.3985, 409.3829, 545.5081, and 547.5237 for squalene, dehydrosqualene, phytoene, and hydrogenated phytoene (lycopersene), respectively. Their MS/MS behaviors were identical to previous reports: *m*/*z* 545 → 137, 81, and 69 for phytoene; *m*/*z* 547 → 137, 121, and 69 for lycopersene; *m*/*z* 409 → 326, 299, 215, and 159 for dehydrosqualene; and *m*/*z* 411 → 329, 287, 259, 231, 149, 109, and 69 for squalene,^34−39^ as depicted in Figure 2 and Figures S1–S4.

In (+) APCI/MS/MS, the fragmentation behavior of squalene, dehydrosqualene, lycopersene, and phytoene is very similar. All four compounds generated ions at *m*/*z* 177.1636, 175.1481, 163.1480, 161.1324, 149.1324, 135.1168, 123.1169, 121.1013, 109.1015, 95.0860, 83.0855, 81.0705, and 69.0706, which were predicted as [C_13_H_21_]^+^, [C_13_H_19_]^+^, [C_12_H_19_]^+^, [C_12_H_17_]^+^, [C_11_H_17_]^+^, [C_10_H_15_]^+^, [C_9_H_15_]^+^, [C_9_H_13_]^+^, [C_8_H_13_]^+^, [C_7_H_11_]^+^, [C_6_H_11_]^+^, [C_6_H_9_]^+^, and [C_5_H_9_]^+^ (*m*/*z* error ≤5 ppm). The major loss in common is 14, indicating the loss of methylene (CH_2_), which fully complies with the fragmentation pattern of the long-chain terpenes.

### 3.2 Comparison of the Yield of Squalene, Dehydrosqualene, and Phytoene *In Vitro* and *In Vivo*

**Figure 3:**
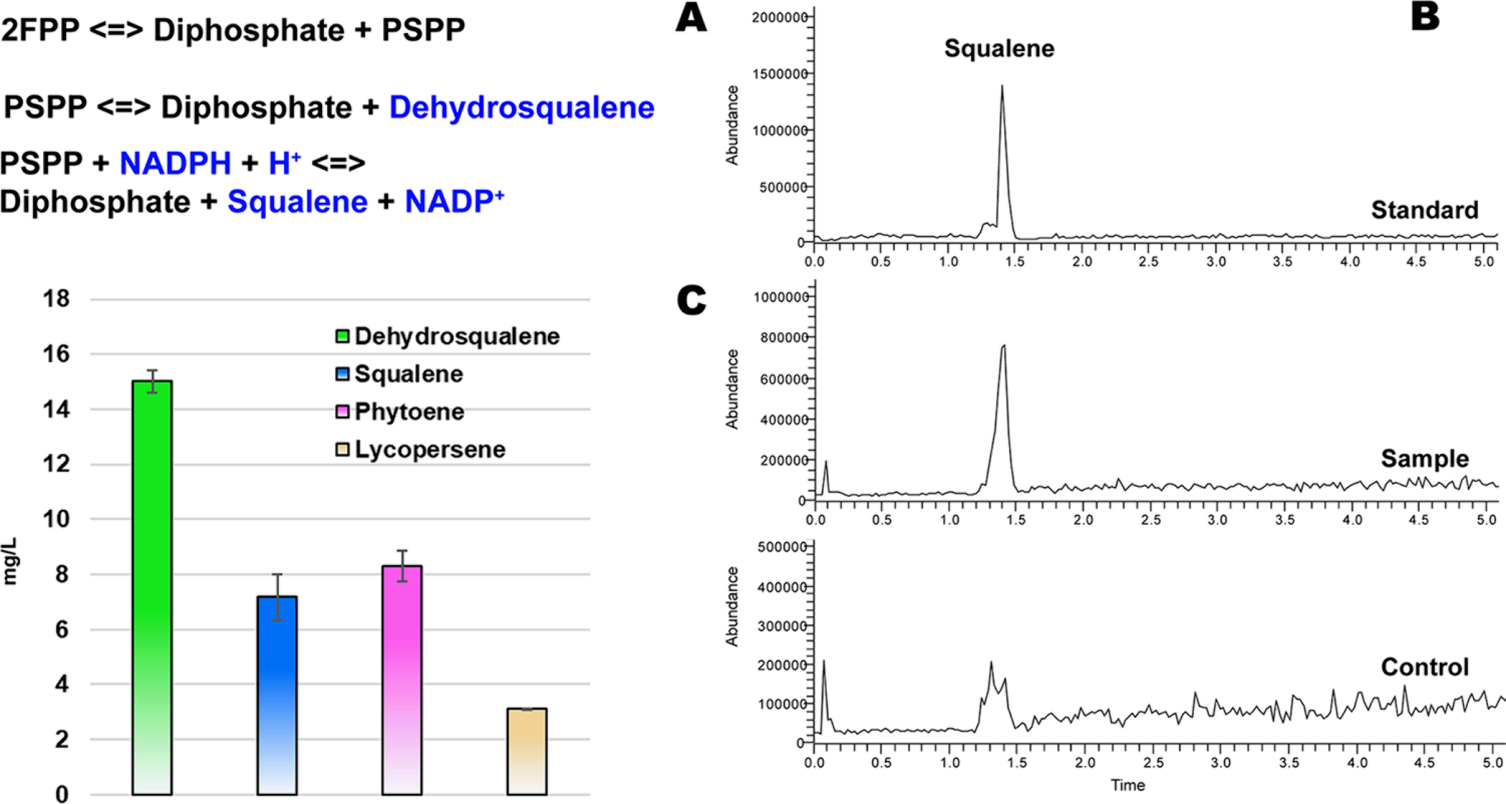
*In vitro* reactions of CrtM analyzed by HPLC-Q-Orbitrap-MS/MS (*n* = 3). (A) Reactions. (B) Chromatograms of the squalene standard, reaction sample, and the reaction control without CrtM. (C) Reaction product concentrations.

Significantly, HPLC-Q-Orbitrap-MS/MS analysis demonstrated that CrtM possesses the ability to employ FPP and GGPP in the presence of NAD(P)H in an *in vitro* setting to synthesize squalene, dehydrosqualene, phytoene, and lycopersene. Notably, when equimolar concentrations of FPP and GGPP were used as substrates in a 15 min reaction, CrtM exhibited catalytic activity, resulting in the production of about 15 mg/L squalene and 3 mg/L lycopersene, thereby highlighting its remarkable catalytic versatility (Figure 3).

Furthermore, intriguing findings from this study revealed that within the same catalytic system, CrtM showed a higher efficacy in utilizing FPP as the substrate, leading to an enhanced yield of squalene over lycopersene. The same situation has also been observed *in vivo*. Without additional NAD(P)H, and also due to the naturally lower GGPP (compared to FPP) levels in bacteria, lycopersene was only found in the BSQS group among all the bacterial groups and exhibited an exceedingly weak signal *in vivo*. This discrepancy could potentially be attributed to the smaller molecular size of FPP, facilitating its entry and affinity in the active pocket.

**Figure 4:**
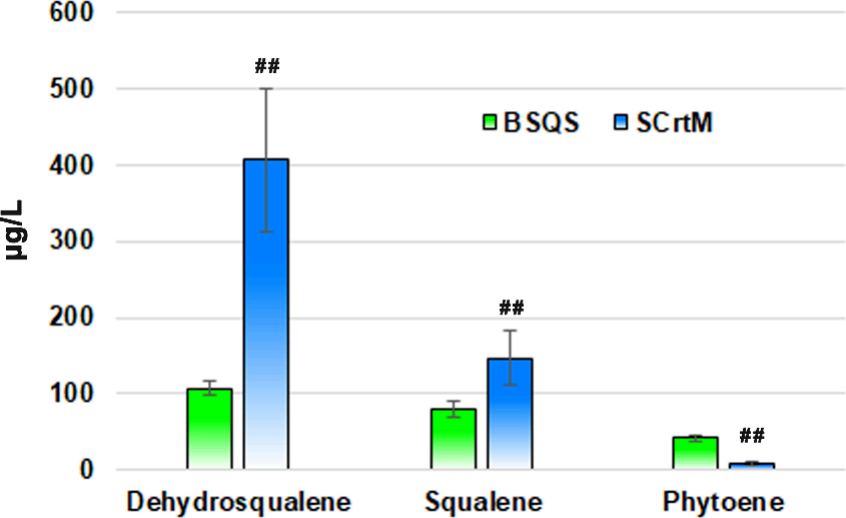
Quantity of squalene, and relative quantity of dehydrosqualene and phytoene in BSQS strain and SCrtM strain (both cultured in TSB). “^##^”, *P* < 0.01. Six biological replicates per group.

Therefore, during the *in vivo* comparison of SSL strains, the quantity of lycopersene was excluded. As shown in Figure 4, the study demonstrates that the introduction of SCrtM or BSQS into wild-type *B. subtilis* 168 causes the production of squalene, dehydrosqualene, and phytoene. The output of squalene, dehydrosqualene, and phytoene are 147, 406, and 7 μg/L in SCrtM strain; and 80, 107, and 41 μg/L in BSQS strain, respectively. Furthermore, as depicted in Figure 4, a clear disparity is observed when comparing the efficiency of FPP utilization between the BSQS and SCrtM strains, resulting in differential terpenoid production, notably squalene (*P* < 0.01). Despite BSQS exhibiting superior selectivity for squalene synthesis, our findings surprisingly indicate the potential of SCrtM as a robust producer of squalene (147 μg/L for SCrtM versus 80 μg/L for BSQS), thereby underscoring its capacity as a promising alternative enzyme for squalene production. Moreover, the study also revealed that both BSQS and SCrtM strains are more inclined to use FPP for the dehydrosqualene synthesis. This preference may arise from the essential involvement of NAD(P)H and H^+^ in the intricate process of squalene biosynthesis.^40,41^

Thus, the findings collectively indicate the remarkable promiscuity of SSL enzymes for their ability not only to catalyze a hydrogen transfer/dehydrogenation reaction to generate squalene or lycopersene during the head-to-head condensation but also to facilitate a dephosphorylation reaction leading to the production of dehydrosqualene or phytoene in the second-step reaction.

**Figure 5:**
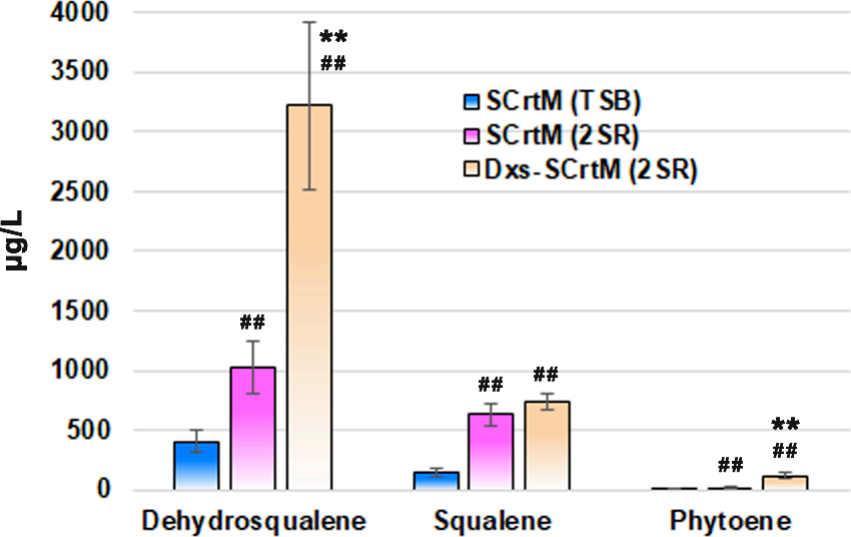
Quantity of squalene, and relative quantity of dehydrosqualene and phytoene in SCrtM strains. SCrtM (TSB), *B. subtilis* 168 with *crtM* cultured in TSB. SCrtM (2SR), *B. subtilis* 168 with *crtM* cultured in 2SR. Dxs-SCrtM (2SR), *B. subtilis* 168 with *crtM* and *dxs* cultured in 2SR. “^##^”, compared to the first group (blue), *P* < 0.01. “**”, compared to the second group (violet), *P* < 0.01. Six biological replicates per group.

To assess the potential of CrtM as a substitute for SQS in squalene production, further investigation was conducted to validate the hypothesis regarding the performance of SCrtM. Results demonstrated that the synthesis efficiency of SCrtM was notably enhanced in the rich medium (2SR), resulting in increased production levels of dehydrosqualene, squalene, and phytoene. Figure 5 provides a graphical representation showing the changes observed, where dehydrosqualene increased from 407 to 1027 μg/L; squalene increased from 147 to 630 μg/L; and phytoene increased from 8 to 17 μg/L. Furthermore, the introduction of upstream *dxs* further augmented the biosynthesis of these compounds, resulting in production levels of 3218, 730, and 120 μg/L for dehydrosqualene, squalene, and phytoene, respectively. Although, compared to the BSQS strain, SCrtM exhibited a distinct preference for dehydrosqualene synthesis in terms of synthesis selectivity (Figure 4), its squalene synthesis efficiency (630 μg/L) was still superior to that of SQS from *B. megaterium* (260 μg/L).^23^ This finding indicates the potential usage of CrtM to replace SQS in squalene biosynthetic cell factories for higher squalene and total squalene-like compound production for food additive, vaccine adjuvant, and cosmetic applications.

Besides, another interesting phenomenon discovered in this study is that, while the up-regulation of *dxs* showed a trend toward increased squalene production, this difference was not statistically significant (*P* = 0.104). This finding aligns with the established understanding that NAD(P)H and H^+^ play vital roles in squalene synthesis.^41^ Despite the nutrient-rich composition of the 2SR medium, no additional supplementation of NAD(P)H and H^+^ was provided. Consequently, enhancing FPP availability through upstream gene up-regulation alone did not yield further improvements in squalene production. We postulate that the main driver to further improve squalene production by SCrtM is its catalytic selectivity. Notably, compared with BSQS (Figure 4), SCrtM exhibits significantly lower selectivity in catalyzing squalene formation under identical conditions. Hence, examining differences in protein sequence and stereo structure between SQS and CrtM, especially their interactions with presqualene diphosphate during the second-step reaction, may offer a fruitful avenue for future investigation.

### 3.3 Compare the Squalene Synthase-Like Enzymes *In Silico*

While extensive studies^26,27,42,43^ have been conducted to understand the mechanisms of SCrtM and HSQS in catalyzing head-to-head condensation, there remain some unanswered questions. In light of this, we explored the possible reasons behind the selective differences between different SSL enzymes in the second-step reaction of head-to-head condensation. To achieve this objective, we not only compared the protein sequence similarity of SSL enzymes but also used PSPP as the binding ligand and discussed the corresponding product changes. The stereostructure comparison is primarily focused on three aspects involving the similarity of stereo structures of SSL enzymes, protein–ligand binding strength, and their spatial positions.

**Figure 6:**
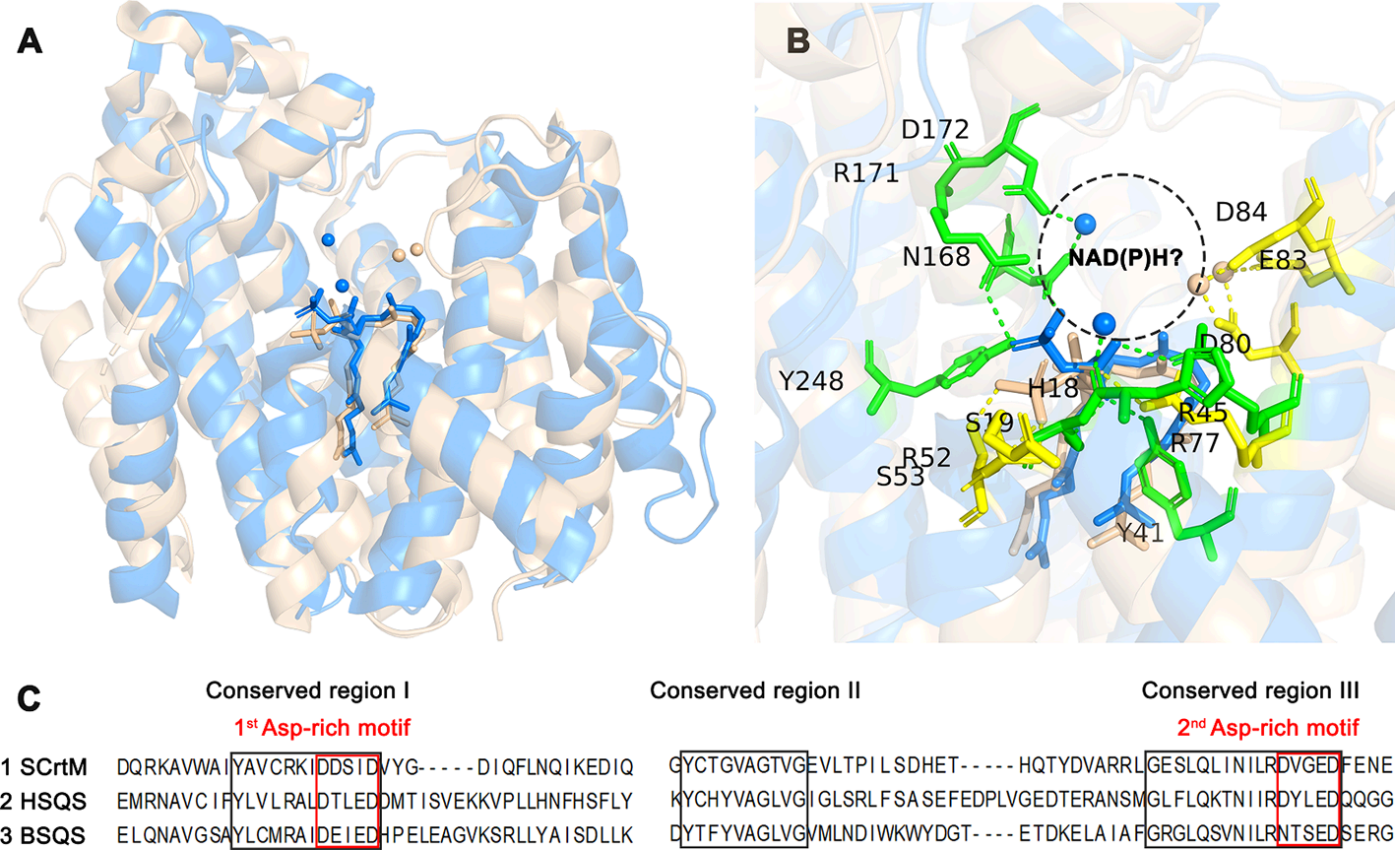
Comparison of SSL enzymes. (A) Structural superposition of SCrtM on HSQS complexed with two PSPP molecules and magnesium. Blue, SCrtM (PDB ID 3NPR); wheat, HSQS (PDB ID 3WEH). (B) Close-up view of the structural superposition of SCrtM on HSQS complexed with two PSPP molecules and magnesium. Blue, SCrtM; wheat, HSQS. The yellow sticks and dashed lines represent the interaction residues and bonds between HSQS and PSPP, while the green sticks and dashed lines represent the corresponding residues and interactions between SCrtM and PSPP (all within a distance of 3.0 Å). The black dashed circle indicates the possible entry and exit position of NAD(P)H and NAD(P)^+^. (C) Sequence alignment of the SCrtM, HSQS, and BSQS.

**Figure 7:**
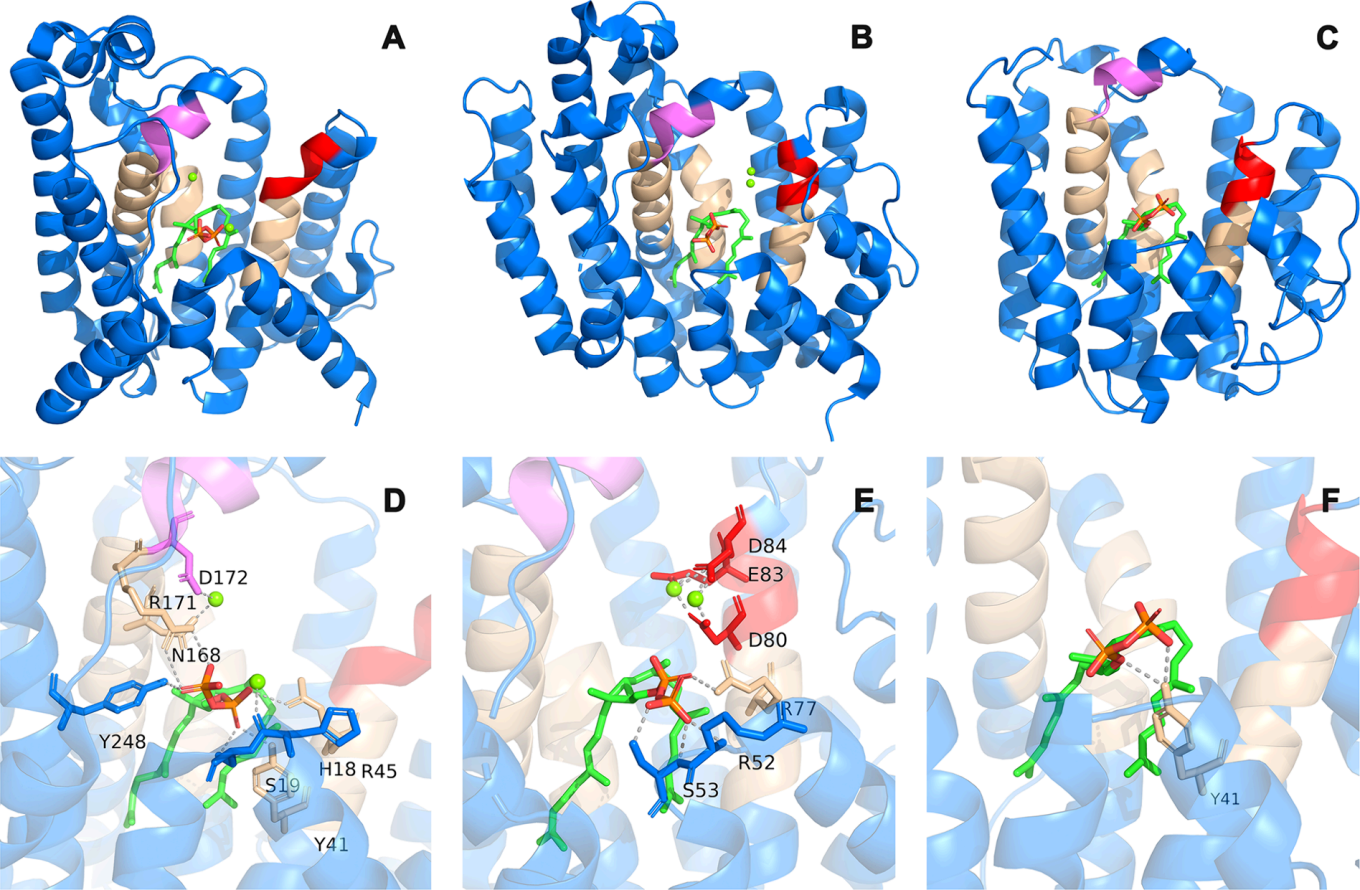
Stereo structures of SCrtM, HSQS, and BSQS. (A) SCrtM complexed with PSPP. (B) HSQS complexed with PSPP. (C) BSQS model complexed with PSPP (model docking result). The wheat parts indicated the conserved regions, and the red and violet parts indicate the first and second Asp-rich regions, respectively. (D–F) Close-up view of the ligand-binding site in panels A, B, and C. The gray dashed lines indicate interaction bonds within a distance of 3.0 Å.

The major comparisons of the SSL enzymes are listed in Figure 6. The comparison of the SCrtM, HSQS, and BSQS is shown in Figure 7A, B, and C, respectively, and the conserved sequences (Figure 6C) were marked in the corresponding positions in these stereo structures. Figure 7D, E, and F are close-ups of the ligand-binding site of Figure 7A, B, and C, respectively.

Based on the EMBOSS Needle sequence comparison, the protein sequence similarity between SCrtM and HSQS was determined to be 38.9% while the similarity between BSQS and HSQS was found to be 43.6% (Table S5). These results provide further support for the functional resemblance observed in both *in vitro* and *in vivo* investigations. Moreover, according to the findings from Liu et al.,^27^ the structure of the HSQS-PSPP-Mg^2+^ complex displays similarities with the previously determined crystal structure of SCrtM with an RMSD value of 2.5 Å between 253 C_α_ atoms, despite having different relative orientations of the pyrophosphate group. This observation is presented in Figure 6A, whereby the stereo structures of HSQS and SCrtM bear striking resemblances even in conserved regions when they bind to PSPP. Additionally, the conformation of PSPP displays remarkable similarity too, particularly for the remaining two-thirds apart from the pyrophosphate group. Besides, we can also observe a similar binding status of PSPP with SCrtM (PDB ID 3NPR), HSQS (PDB ID 3WEH), and BSQS (model docking result) in Figure 7A, B, and C, respectively.

In addition to exploring the similarity of stereo structures of SSL enzymes, we further analyzed the binding bonds between these enzymes and the ligand. In protein–ligand interactions, hydrogen bond interactions and van der Waals forces are crucial factors that determine the binding strength. We mainly focused on analyzing the hydrogen bond binding situation between SSL enzymes and PSPP, as hydrogen bond interactions (usually between 5 and 40 kJ/mol) often play a dominant role in protein–ligand interactions due to their greater energy contribution compared to van der Waals forces (usually ≤4 kJ/mol). Our analysis revealed that strong hydrogen bonds or coordination bonds (with Mg^2+^) between SSL enzymes and PSPP were primarily concentrated near the phosphate groups and magnesium ions (Figure 7D–F), which is consistent with previous studies showing the essential role of these components in the head-to-head condensation process,^27,43^ including the participation of magnesium ions in dephosphorylation. Therefore, the synthetic selectivity of SSL enzymes in the second step of the reaction may be significantly influenced by the conformation of phosphate groups and the positions of magnesium ions.

As reported by Malwal et al.,^43^ the location where NAD(P)H and NAD(P)^+^ enter and exit the protein cavity was identified as an essential influencing factor for squalene biosynthesis, which showed that they should only enter and exit from the front of the SSL enzyme structure. Upon examining Figure 6B, we observed that several major residues in HSQS that bind to magnesium ions and phosphate groups at close distances (≤3.0 Å) not only have smaller sizes but also pull magnesium ions and PSPP in opposite directions (yellow part in Figure 6B), which creates a larger entrance in the protein for NAD(P)H. Conversely, the larger residues in SCrtM that bind to PSPP bring magnesium ions and phosphate groups closer together near the pocket’s opening. This may result in significant differences in the entry and exit of NAD(P)H and NAD(P)^+^ into the pocket, as the steric hindrance formed by HSQS with PSPP and magnesium ions is significantly less than that in SCrtM. Therefore, we propose that this situation may be the primary reason for SCrtM’s low NAD(P)H binding rate, leading to its tendency toward synthesizing dehydrosqualene instead of squalene during the catalysis of head-to-head condensation. Based on this finding, possible further work may be directed to selective mutation to improve the squalene synthesis efficiency of SCrtM for higher squalene yield.

In this study, we report for the first time that CrtM from *S. aureus* has the natural ability to produce not only dehydrosqualene but also squalene and phytoene both *in vitro* or in *B. subtilis*. Moreover, we observed the same products in *B. subtilis* strains that contain SQS, indicating the promiscuity of the SSL enzymes. Interestingly, comparative analyses revealed that the CrtM from *S. aureus* could even produce more squalene than the SQS from *B. megaterium* in *B. subtilis* 168, which indicates the possibility of CrtM replacing the SQS in different cell factories for more squalene production. Additionally, by adjusting the medium to a nutrition-rich medium or inserting *dxs* to upregulate the upstream donors, CrtM was able to greatly increase the yield of squalene, which provides further evidence for its potential in squalene biosynthesis. Furthermore, the stereo structure comparison provided insights into the molecular basis of ligand-binding mode and substrate specificity, which could guide the further mutagenesis to increase the squalene biosynthetic yield of CrtM.

## Supporting Information Available

Plasmids used in this study, strains used in this study, primers used in this study, squalene standard curve, docking information, sequence comparison, and compound MS2 profiles (PDF)

## Abbreviations

CrtM: dehydrosqualene synthase
SQS: squalene synthase
SSL: squalene synthase-like
FPP: farnesyl diphosphate
PSPP: presqualene diphosphate
GGPP: geranylgeranyl diphosphate

## References

[ref1] Micera M.; Botto A.; Geddo F.; Antoniotti S.; Bertea C. M.; Levi R.; Gallo M. P.; Querio G. Squalene: More than a Step toward Sterols. Antioxidants 2020, 9 (8), 688 10.3390/antiox9080688. 32748847 PMC7464659

[ref2] Shen J.; Liu Y.; Wang X.; Bai J.; Lin L.; Luo F.; Zhong H. A Comprehensive Review of Health-Benefiting Components in Rapeseed Oil. Nutrients 2023, 15 (4), 999 10.3390/nu15040999. 36839357 PMC9962526

[ref3] Renda G.; Gökkaya İ.; Şöhretoğlu D. Immunomodulatory Properties of Triterpenes. Phytochem Rev. 2022, 21 (2), 537–563. 10.1007/s11101-021-09785-x. 34812259 PMC8600492

[ref4] Mendes A.; Azevedo-Silva J.; Fernandes J. C. From Sharks to Yeasts: Squalene in the Development of Vaccine Adjuvants. Pharmaceuticals 2022, 15 (3), 265 10.3390/ph15030265. 35337064 PMC8951290

[ref5] Song X.; Wang X.; Tan Y.; Feng Y.; Li W.; Cui Q. High Production of Squalene Using a Newly Isolated Yeast-like Strain Pseudozyma Sp. SD301. Journal of agricultural and food chemistry 2015, 63 (38), 8445–8451. 10.1021/acs.jafc.5b03539. 26350291

[ref6] Chang M.-H.; Kim H.-J.; Jahng K.-Y.; Hong S.-C. The Isolation and Characterization of Pseudozyma Sp. JCC 207, a Novel Producer of Squalene. Applied microbiology and biotechnology 2008, 78, 963–972. 10.1007/s00253-008-1395-4. 18299826

[ref7] Liu C.-I.; Liu G. Y.; Song Y.; Yin F.; Hensler M. E.; Jeng W.-Y.; Nizet V.; Wang A. H.-J.; Oldfield E. A Cholesterol Biosynthesis Inhibitor Blocks Staphylococcus aureus Virulence. Science 2008, 319 (5868), 1391–1394. 10.1126/science.1153018. 18276850 PMC2747771

[ref8] Umeno D.; Tobias A. V.; Arnold F. H. Evolution of the C30 Carotenoid Synthase CrtM for Function in a C40 Pathway. J. Bacteriol. 2002, 184 (23), 6690–6699. 10.1128/JB.184.23.6690-6699.2002. 12426357 PMC135437

[ref9] Ku B.; Jeong J.-C.; Mijts B. N.; Schmidt-Dannert C.; Dordick J. S. Preparation, Characterization, and Optimization of an in Vitro C30 Carotenoid Pathway. Applied and environmental microbiology 2005, 71 (11), 6578–6583. 10.1128/AEM.71.11.6578-6583.2005. 16269684 PMC1287715

[ref10] Pelz A.; Wieland K.-P.; Putzbach K.; Hentschel P.; Albert K.; Gotz F. Structure and Biosynthesis of Staphyloxanthin from Staphylococcus aureus. J. Biol. Chem. 2005, 280 (37), 32493–32498. 10.1074/jbc.M505070200. 16020541

[ref11] Thapa H. R.; Naik M. T.; Okada S.; Takada K.; Molnár I.; Xu Y.; Devarenne T. P. A Squalene Synthase-like Enzyme Initiates Production of Tetraterpenoid Hydrocarbons in Botryococcus braunii Race L. Nat. Commun. 2016, 7 (1), 11198 10.1038/ncomms11198. 27050299 PMC4823828

[ref12] Xue L.; Chen Y. Y.; Yan Z.; Lu W.; Wan D.; Zhu H. Staphyloxanthin: A Potential Target for Antivirulence Therapy. Infect. Drug Resist. 2019, 2151–2160. 10.2147/IDR.S193649. 31410034 PMC6647007

[ref13] Elmesseri R. A.; Saleh S. E.; Elsherif H. M.; Yahia I. S.; Aboshanab K. M. Staphyloxanthin as a Potential Novel Target for Deciphering Promising Anti-Staphylococcus aureus Agents. Antibiotics 2022, 11 (3), 298 10.3390/antibiotics11030298. 35326762 PMC8944557

[ref14] Elmesseri R. A.; Saleh S. E.; Ghobish S. A.; Majrashi T. A.; Elsherif H. M.; Aboshanab K. M. Diclofenac and Meloxicam Exhibited Anti-Virulence Activities Targeting Staphyloxanthin Production in Methicillin-Resistant Staphylococcus aureus. Antibiotics 2023, 12 (2), 277 10.3390/antibiotics12020277. 36830188 PMC9951919

[ref15] Umeno D.; Arnold F. H. Evolution of a Pathway to Novel Long-Chain Carotenoids. J. Bacteriol. 2004, 186 (5), 1531–1536. 10.1128/JB.186.5.1531-1536.2004. 14973014 PMC344396

[ref16] Furubayashi M.; Saito K.; Umeno D. Evolutionary Analysis of the Functional Plasticity of Staphylococcus aureus C30 Carotenoid Synthase. J. Biosci. Bioeng. 2014, 117 (4), 431–436. 10.1016/j.jbiosc.2013.10.003. 24216462

[ref17] Furubayashi M.; Ikezumi M.; Takaichi S.; Maoka T.; Hemmi H.; Ogawa T.; Saito K.; Tobias A. V.; Umeno D. A Highly Selective Biosynthetic Pathway to Non-Natural C50 Carotenoids Assembled from Moderately Selective Enzymes. Nat. Commun. 2015, 6 (1), 7534 10.1038/ncomms8534. 26168783 PMC4510654

[ref18] Li L.; Furubayashi M.; Hosoi T.; Seki T.; Otani Y.; Kawai-Noma S.; Saito K.; Umeno D. Construction of a Nonnatural C60 Carotenoid Biosynthetic Pathway. ACS Synth. Biol. 2019, 8 (3), 511–520. 10.1021/acssynbio.8b00385. 30689939

[ref19] Xue D.; Abdallah I. I.; de Haan I. E.; Sibbald M. J.; Quax W. J. Enhanced C 30 Carotenoid Production in Bacillus subtilis by Systematic Overexpression of MEP Pathway Genes. Appl. Microbiol. Biotechnol. 2015, 99, 5907–5915. 10.1007/s00253-015-6531-3. 25851715 PMC4480331

[ref20] Yoshida K.; Ueda S.; Maeda I. Carotenoid Production in Bacillus subtilis Achieved by Metabolic Engineering. Biotechnology letters 2009, 31, 1789–1793. 10.1007/s10529-009-0082-6. 19618272

[ref21] Radeck J.; Kraft K.; Bartels J.; Cikovic T.; Dürr F.; Emenegger J.; Kelterborn S.; Sauer C.; Fritz G.; Gebhard S. The Bacillus BioBrick Box: Generation and Evaluation of Essential Genetic Building Blocks for Standardized Work with Bacillus subtilis. J. Biol. Eng. 2013, 7 (1), 1–17. 10.1186/1754-1611-7-29. 24295448 PMC4177231

[ref22] You C.; Zhang X.-Z.; Zhang Y.-H. P. Simple Cloning via Direct Transformation of PCR Product (DNA Multimer) to Escherichia coli and Bacillus subtilis. Applied and environmental microbiology 2012, 78 (5), 1593–1595. 10.1128/AEM.07105-11. 22194286 PMC3294473

[ref23] Song Y.; Guan Z.; van Merkerk R.; Pramastya H.; Abdallah I. I.; Setroikromo R.; Quax W. J. Production of Squalene in Bacillus subtilis by Squalene Synthase Screening and Metabolic Engineering. Journal of agricultural and food chemistry 2020, 68 (15), 4447–4455. 10.1021/acs.jafc.0c00375. 32208656 PMC7168599

[ref24] Huang H.; Chu C.-L.; Chen L.; Shui D. Evaluation of Potential Inhibitors of Squalene Synthase Based on Virtual Screening and in Vitro Studies. Computational Biology and Chemistry 2019, 80, 390–397. 10.1016/j.compbiolchem.2019.04.008. 31125877

[ref25] Kourounakis A. P.; Matralis A. N.; Nikitakis A. Design of More Potent squalene Synthase Inhibitors with Multiple Activities. Bioorganic & medicinal chemistry 2010, 18 (21), 7402–7412. 10.1016/j.bmc.2010.09.008. 20888243

[ref26] Lin F.-Y.; Liu C.-I.; Liu Y.-L.; Zhang Y.; Wang K.; Jeng W.-Y.; Ko T.-P.; Cao R.; Wang A. H.-J.; Oldfield E. Mechanism of Action and Inhibition of Dehydrosqualene Synthase. Proc. Natl. Acad. Sci. U. S. A. 2010, 107 (50), 21337–21342. 10.1073/pnas.1010907107. 21098670 PMC3003041

[ref27] Liu C.-I.; Jeng W.-Y.; Chang W.-J.; Shih M.-F.; Ko T.-P.; Wang A.-J. Structural Insights into the Catalytic Mechanism of Human squalene Synthase. Acta Crystallographica Section D: Biological Crystallography 2014, 70 (2), 231–241. 10.1107/S1399004713026230. 24531458

[ref28] Webb B.; Sali A. Comparative Protein Structure Modeling Using MODELLER. Curr. Protoc. Bioinf. 2016, 54 (1), 5.6. 1–5.6. 37. 10.1002/cpbi.3. PMC5031415 27322406

[ref29] Zimmermann L.; Stephens A.; Nam S.-Z.; Rau D.; Kübler J.; Lozajic M.; Gabler F.; Söding J.; Lupas A. N.; Alva V. A Completely Reimplemented MPI Bioinformatics Toolkit with a New HHpred Server at Its Core. Journal of molecular biology 2018, 430 (15), 2237–2243. 10.1016/j.jmb.2017.12.007. 29258817

[ref30] Gabler F.; Nam S.-Z.; Till S.; Mirdita M.; Steinegger M.; Söding J.; Lupas A. N.; Alva V. Protein Sequence Analysis Using the MPI Bioinformatics Toolkit. Current Protocols in Bioinformatics 2020, 72 (1), e108 10.1002/cpbi.108. 33315308

[ref31] Liu Y.; Yang X.; Gan J.; Chen S.; Xiao Z.-X.; Cao Y. CB-Dock2: Improved Protein–Ligand Blind Docking by Integrating Cavity Detection, Docking and Homologous Template Fitting. Nucleic Acids Res. 2022, 50 (W1), W159–W164. 10.1093/nar/gkac394. 35609983 PMC9252749

[ref32] Eberhardt J.; Santos-Martins D.; Tillack A. F.; Forli S. AutoDock Vina 1.2. 0: New Docking Methods, Expanded Force Field, and Python Bindings. J. Chem. Inf. Model. 2021, 61 (8), 3891–3898. 10.1021/acs.jcim.1c00203. 34278794 PMC10683950

[ref33] Crean R. M.; Gardner J. M.; Kamerlin S. C. Harnessing Conformational Plasticity to Generate Designer Enzymes. J. Am. Chem. Soc. 2020, 142 (26), 11324–11342. 10.1021/jacs.0c04924. 32496764 PMC7467679

[ref34] Zarrouk W.; Carrasco-Pancorbo A.; Segura-Carretero A.; Fernandez-Gutierrez A.; Zarrouk M. Exploratory Characterization of the Unsaponifiable Fraction of Tunisian Virgin Olive Oils by a Global Approach with HPLC-APCI-IT MS/MS Analysis. Journal of agricultural and food chemistry 2010, 58 (10), 6418–6426. 10.1021/jf100024c. 20438134

[ref35] Rivera S. M.; Christou P.; Canela-Garayoa R. Identification of Carotenoids Using Mass Spectrometry. Mass Spectrom. Rev. 2014, 33 (5), 353–372. 10.1002/mas.21390. 24178708

[ref36] Schex R.; Lieb V. M.; Jiménez V. M.; Esquivel P.; Schweiggert R. M.; Carle R.; Steingass C. B. HPLC-DAD-APCI/ESI-MSn Analysis of Carotenoids and α-Tocopherol in Costa Rican Acrocomia aculeata Fruits of Varying Maturity Stages. Food Research International 2018, 105, 645–653. 10.1016/j.foodres.2017.11.041. 29433258

[ref37] López G.-D.; Suesca E.; Álvarez-Rivera G.; Rosato A. E.; Ibáñez E.; Cifuentes A.; Leidy C.; Carazzone C. Carotenogenesis of Staphylococcus aureus: New Insights and Impact on Membrane Biophysical Properties. Biochimica et Biophysica Acta (BBA)-Molecular and Cell Biology of Lipids 2021, 1866 (8), 158941 10.1016/j.bbalip.2021.158941. 33862238

[ref38] Arumugam S.; Ramessh C.; Kaliappan G. K.; Govindhan R.; Prakasam S. B.; Murugan S.; Pandian S.; Asgar E.; Ravi P. Lycopersene: A Review on Extraction, Identification and Purification and Applications. Chemical Biology & Drug Design 2023, 101 (1), 158–174. 10.1111/cbdd.14158. 36377692

[ref39] Qureshi A. A.; Barnes F. J.; Semmler E. J.; Porter J. W. Biosynthesis of Prelycopersene Pyrophosphate and Lycopersene by squalene Synthetase. J. Biol. Chem. 1973, 248 (8), 2755–2767. 10.1016/S0021-9258(19)44071-4. 4144543

[ref40] Blagg B. S.; Jarstfer M. B.; Rogers D. H.; Poulter C. D. Recombinant squalene Synthase. A Mechanism for the Rearrangement of Presqualene Diphosphate to squalene. J. Am. Chem. Soc. 2002, 124 (30), 8846–8853. 10.1021/ja020411a. 12137537

[ref41] Jarstfer M. B.; Zhang D.-L.; Poulter C. D. Recombinant squalene Synthase. Synthesis of Non-Head-to-Tail Isoprenoids in the Absence of NADPH. J. Am. Chem. Soc. 2002, 124 (30), 8834–8845. 10.1021/ja020410i. 12137536

[ref42] Oldfield E.; Lin F.-Y. Terpene Biosynthesis: Modularity Rules. Angew. Chem., Int. Ed. 2012, 51 (5), 1124–1137. 10.1002/anie.201103110. PMC3769779 22105807

[ref43] Malwal S. R.; Gao J.; Hu X.; Yang Y.; Liu W.; Huang J.-W.; Ko T.-P.; Li L.; Chen C.-C.; O’Dowd B.; Khade R. L.; Zhang Y.; Zhang Y.; Oldfield E.; Guo R.-T. Catalytic Role of Conserved Asparagine, Glutamine, Serine, and Tyrosine Residues in Isoprenoid Biosynthesis Enzymes. ACS Catal. 2018, 8 (5), 4299–4312. 10.1021/acscatal.8b00543. 30345154 PMC6193494

