# Supplementary material for: The Promiscuity of Squalene Synthase-Like Enzyme: Dehydrosqualene Synthase, a Natural Squalene Hyperproducer?
Source: J Agric Food Chem. 2024 Feb 5;72(6):3017–24. doi: 10.1021/acs.jafc.3c05770 (PMC10870770; doi:10.1021/acs.jafc.3c05770)
Supplement: Supplementary file 1 — jf3c05770_si_001.pdf [file jf3c05770_si_001.pdf]

Supplementary Material

The Promiscuity of Squalene Synthase-like Enzyme: Dehydrosqualene Synthase, a Natural Squalene Hyperproducer?

Zheng Guan[1#], Yafeng Song[1,3#], Marcel de Vries[2], Hjalmar Permentier[2], Pieter Tepper[1], Ronald van Merkerk[1], Rita Setroikromo[1], Wim J. Quax[1*]

[1] Department of Chemical and Pharmaceutical Biology, Groningen Research Institute of Pharmacy, University of Groningen, Groningen, 9713 AV, The Netherlands

[2] Interfaculty Mass Spectrometry Center, Groningen Research Institute of Pharmacy, University of Groningen, Groningen, 9713 AV, The Netherlands

[3] Guangdong Provincial Key Laboratory of Microbial Culture Collection and Application, State Key Laboratory of Applied Microbiology Southern China, Institute of Microbiology, Guangdong Acadamy of Sciences, Guangzhou, 510070, China

*** Correspondence:**
Wim J. Quax
*E-mail: w.j.quax@rug.nl
Telephone: +31 50 36 32558

Supplementary Tables and Figures

Table S1 Plasmids and bacterial strains used in this study.

Table S2 Primers used to obtain the needed genes.

Table S3 Squalene standard curve.

Table S4 Docking information.

Table S5 Protein sequence alignments.

Figure S1 Phytoene MS2 profile (CrtM product, *in vitro*)

Figure S2 Lycopersene MS2 profile (CrtM product, *in vitro*)

Figure S3 Dehydrosqualene MS2 profile (CrtM product, *in vitro*)

Figure S4 Squalene MS2 profile (CrtM product, *in vitro*)

Table S1 Plasmids and bacterial strains used in this study.

Plasmid	Genotype and/or relevant characteristics	Source/Reference
pHY300PLK	*B. subtilis* and *E. coli* shuttle vector; ori-pACYC177; ori-pAMα1; Tc^R ; Amp^R	[1]
pHY-BSQS	pHY300PLK derivative, squalene synthase originated from *Bacillus megaterium*	This work
pHY-CrtMN	pHY300PLK derivative, *crtM* and *crtN* genes originated from *Staphylococcus aureus*	This work
pHY-CrtM	pHY300PLK derivative, *crtM* gene originated from *Staphylococcus aureus*	This work
pHCMC04G	*B. subtilis* and *E. coli* shuttle vector; ori-pBR322; ori-pBS72; P_{xylA} xylose-inducible promoter; Cm^R ; Amp^R	[2]
pHCMC04G-S	pHCMC04G derivative, *dxs* gene originated from *Bacillus subtilis*	This work
**Bacterial strain**	**Genotype and/or relevant characteristics**	**Source/Reference**
B. subtilis 168	*trpC2*	[3,4]
E. coli DH5α	F^- *end*A1 *hsd*R17 (r_k^-, m_k^+) *sup*E44 *thi*$^-$ 1 λ^-*rec*A1 *gyr*A96 *rel*A1 φ80d*lac*ZΔM15	Bethesda Research Laboratories 1986
BpHY	*B. subtilis* 168 derivative, pHY300PLK, Tc^R,	This work
BC	*B. subtilis* 168 derivative, pHCMC04G, Cm^R	This work
BSQS	*B. subtilis* 168 derivative, pHY-BSQS, Tc^R	This work
ScrtMN	*B. subtilis* 168 derivative, pHY-CrtMN, Tc^R	This work
ScrtM	*B. subtilis* 168 derivative, pHY-CrtM, Tc^R	This work
S-ScrtM	*B. subtilis* 168 derivative, pHCMC04G-S, pHY-CrtM, Cm^R, Tc^R	This work

Table S2 Primers used to obtain the needed genes.

Name	Sequence
BSQS-F	GACAAATGGTCCAAACTAGTGATAAGAGGAGGAGAAATATGAGCGTTCCGAATAAACTGCGCG
BSQS-R	CATTTCCCCCTTTGATTTTTAGATTCAGTGATGATGATGATGATGCATATCGACGACTTCATTGACTG
SCrtMN-F	GCGGATCCGTAAGAGAGGACTAGTATGAC
SCrtMN-R	CGCCGTCGACCGTTATGTTCAACAG

Table S3 Squalene standard curve.

Conc. (μg/ml)	1	4	16	64	256	512
Peak	1919992.5	6855682.6	22719952.4	112365424	475391319	914624414
Regression eq.	$y = 6E-07x + 0.2423$, $R^2 = 0.9996$					

Table S4 Docking information.

Enzyme	Vina score (affinity, kcal/mol)	Center (x, y, z)	Docking size (x, y, z)	Contact residues
BSQS model (GenBank: ADF40697.1, Figure 4C, 4G)	−9.3	23, 47, 221	26, 26, 26	THR18 SER19 THR21 PHE22 VAL37 ALA40 TYR41 MET44 ARG45 ASP48 MET121 VAL148 ALA149 VAL152 GLY153 MET155 LEU156 ASN157 TRP160 ALA173 PHE176 GLY177 LEU180 GLN181 ASN184 TYR223 PHE235 CYS236

* Docking ligand: PSPP

* Docking tool [5,6]

Table S5 Protein sequence alignments.

# Program: needle<br># -gapopen 10.0<br># -gapextend 0.5<br># -endopen 10.0<br># -endextend 0.5<br># -aformat3 pair<br># Align_format: pair<br># Aligned_sequences: 2<br># 1: HSQS<br># 2: SCrtM<br># Matrix: EBLOSUM40<br># Gap_penalty: 10.0<br># Extend_penalty: 0.5<br># Length: 386<br># Identity: 84/386 (21.8%)<br># Similarity: 150/386 (38.9%)<br># Gaps: 136/386 (35.2%)<br># Score: 392.0	# Program: needle<br># -gapopen 10.0<br># -gapextend 0.5<br># -endopen 10.0<br># -endextend 0.5<br># -aformat3 pair<br># Align_format: pair<br># Aligned_sequences: 2<br># 1: HSQS<br># 2: BSQS<br># Matrix: EBLOSUM40<br># Gap_penalty: 10.0<br># Extend_penalty: 0.5<br># Length: 358<br># Identity: 86/358 (24.0%)<br># Similarity: 156/358 (43.6%)<br># Gaps: 101/358 (28.2%)<br># Score: 481.5

Figure S1 Phytoene MS2 profile (CrtM product, *in vitro*)

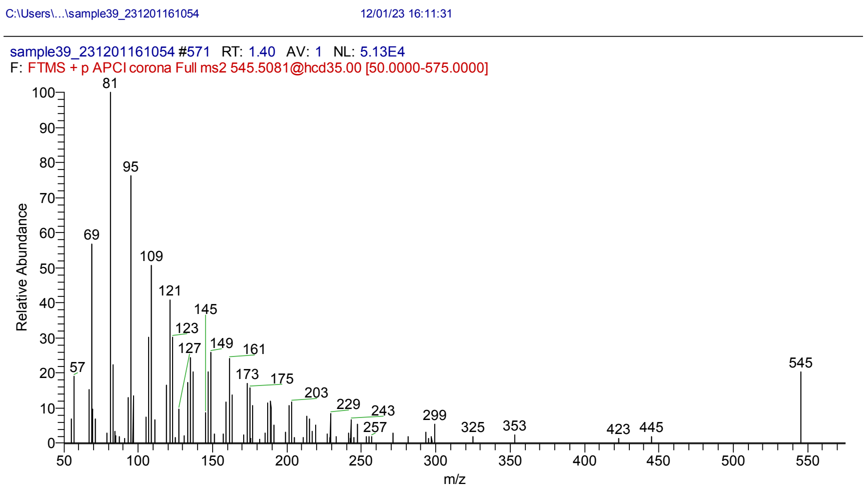

Figure S2 Lycopersene MS2 profile (CrtM product, *in vitro*)

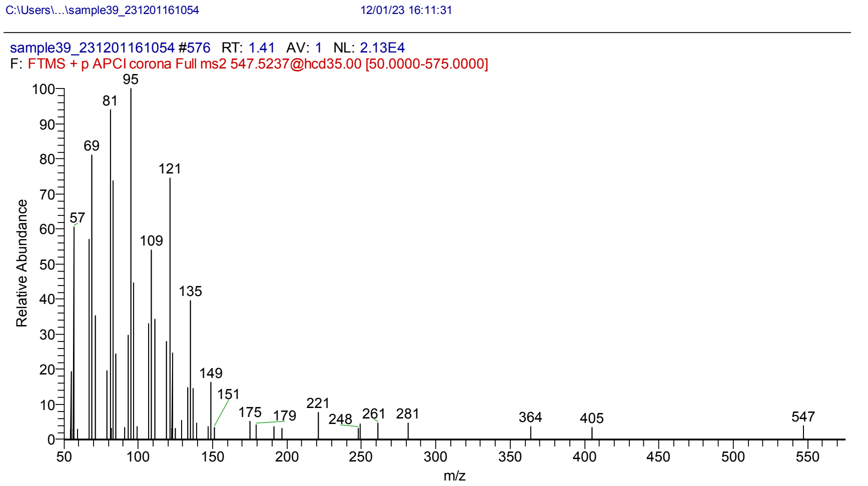

Figure S3 Dehydrosqualene MS2 profile (CrtM product, *in vitro*)

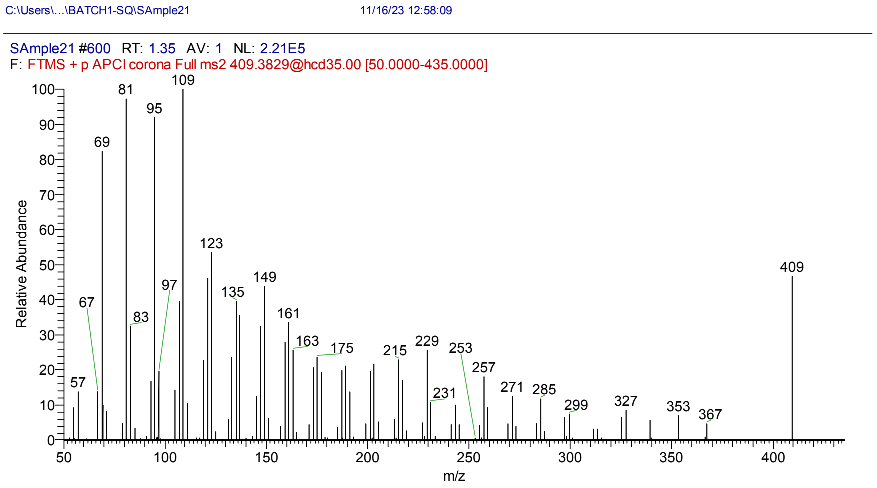

Figure S4 Squalene MS2 profile (CrtM product, *in vitro*)

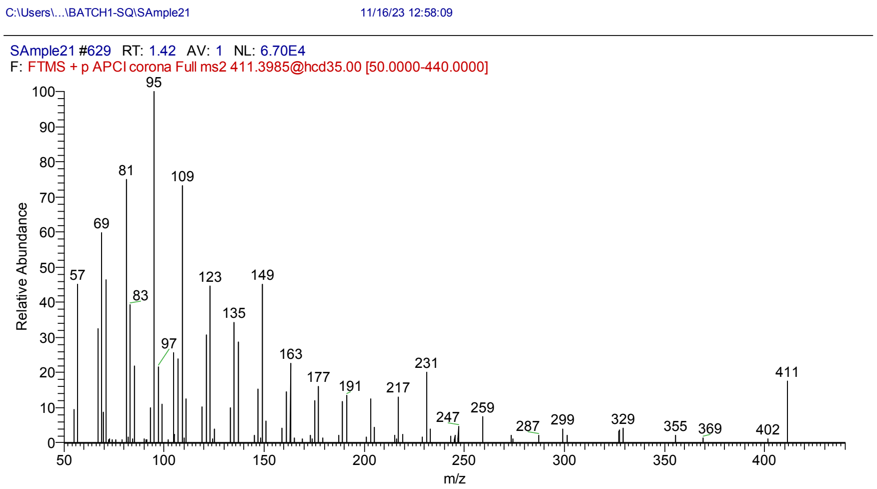

References

(1) Yoshida, K.; Ueda, S.; Maeda, I. Carotenoid Production in Bacillus Subtilis Achieved by Metabolic Engineering. *Biotechnology letters* **2009**, *31*, 1789–1793. https://doi.org/10.1007/s10529-009-0082-6.

(2) Xue, D.; Abdallah, I. I.; de Haan, I. E.; Sibbald, M. J.; Quax, W. J. Enhanced C 30 Carotenoid Production in Bacillus Subtilis by Systematic Overexpression of MEP Pathway Genes. *Applied Microbiology and Biotechnology* **2015**, *99*, 5907–5915. https://doi.org/10.1007/s00253-015-6531-3.

(3) Kunst, F.; Ogasawara, N.; Moszer, I.; Albertini, A. M.; Alloni, G.; Azevedo, V.; Bertero, M. G.; Bessières, P.; Bolotin, A.; Borchert, S.; Borriss, R.; Boursier, L.; Brans, A.; Braun, M.; Brignell, S. C.; Bron, S.; Brouillet, S.; Bruschi, C. V.; Caldwell, B.; Capuano, V.; Carter, N. M.; Choi, S. K.; Cordani, J. J.; Connerton, I. F.; Cummings, N. J.; Daniel, R. A.; Denziot, F.; Devine, K. M.; Düsterhöft, A.; Ehrlich, S. D.; Emmerson, P. T.; Entian, K. D.; Errington, J.; Fabret, C.; Ferrari, E.; Foulger, D.; Fritz, C.; Fujita, M.; Fujita, Y.; Fuma, S.; Galizzi, A.; Galleron, N.; Ghim, S. Y.; Glaser, P.; Goffeau, A.; Golightly, E. J.; Grandi, G.; Guiseppi, G.; Guy, B. J.; Haga, K.; Haiech, J.; Harwood, C. R.; Hènaut, A.; Hilbert, H.; Holsappel, S.; Hosono, S.; Hullo, M. F.; Itaya, M.; Jones, L.; Joris, B.; Karamata, D.; Kasahara, Y.; Klaerr-Blanchard, M.; Klein, C.; Kobayashi, Y.; Koetter, P.; Koningstein, G.; Krogh, S.; Kumano, M.; Kurita, K.; Lapidus, A.; Lardinois, S.; Lauber, J.; Lazarevic, V.; Lee, S. M.; Levine, A.; Liu, H.; Masuda, S.; Mauël, C.; Médigue, C.; Medina, N.; Mellado, R. P.; Mizuno, M.; Moestl, D.; Nakai, S.; Noback, M.; Noone, D.; O'Reilly, M.; Ogawa, K.; Ogiwara, A.; Oudega, B.; Park, S. H.; Parro, V.; Pohl, T. M.; Portelle, D.; Porwollik, S.; Prescott, A. M.; Presecan, E.; Pujic, P.; Purnelle, B.; Rapoport, G.; Rey, M.; Reynolds, S.; Rieger, M.; Rivolta, C.; Rocha, E.; Roche, B.; Rose, M.; Sadaie, Y.; Sato, T.; Scanlan, E.; Schleich, S.; Schroeter, R.; Scoffone, F.; Sekiguchi, J.; Sekowska, A.; Seror, S. J.;

Serror, P.; Shin, B. S.; Soldo, B.; Sorokin, A.; Tacconi, E.; Takagi, T.; Takahashi, H.; Takemaru, K.; Takeuchi, M.; Tamakoshi, A.; Tanaka, T.; Terpstra, P.; Togoni, A.; Tosato, V.; Uchiyama, S.; Vandebol, M.; Vannier, F.; Vassarotti, A.; Viari, A.; Wambutt, R.; Wedler, H.; Weitzenegger, T.; Winters, P.; Wipat, A.; Yamamoto, H.; Yamane, K.; Yasumoto, K.; Yata, K.; Yoshida, K.; Yoshikawa, H. F.; Zumstein, E.; Yoshikawa, H.; Danchin, A. The Complete Genome Sequence of the Gram-Positive Bacterium Bacillus Subtilis. *Nature* **1997**, *390* (6657), 249–256. https://doi.org/10.1038/36786.

(4) Barbe, V.; Cruveiller, S.; Kunst, F.; Lenoble, P.; Meurice, G.; Sekowska, A.; Vallenet, D.; Wang, T.; Moszer, I.; Médigue, C.; Danchin, A. From a Consortium Sequence to a Unified Sequence: The Bacillus Subtilis 168 Reference Genome a Decade Later. *Microbiology (Reading)* **2009**, *155* (Pt 6), 1758–1775. https://doi.org/10.1099/mic.0.027839-0.

(5) Eberhardt, J.; Santos-Martins, D.; Tillack, A. F.; Forli, S. AutoDock Vina 1.2. 0: New Docking Methods, Expanded Force Field, and Python Bindings. *Journal of chemical information and modeling* **2021**, *61* (8), 3891–3898. https://doi.org/10.1021/acs.jcim.1c00203.

(6) Liu, Y.; Yang, X.; Gan, J.; Chen, S.; Xiao, Z.-X.; Cao, Y. CB-Dock2: Improved Protein–Ligand Blind Docking by Integrating Cavity Detection, Docking and Homologous Template Fitting. *Nucleic Acids Research* **2022**, *50* (W1), W159–W164. https://doi.org/10.1093/nar/gkac394.
